# Risk of *Aedes*-borne diseases in and around the Tanzanian seaport of Tanga despite community members being more concerned about malaria

**DOI:** 10.1186/s13071-024-06586-x

**Published:** 2024-12-18

**Authors:** Amri S. Abas, Alfred J. Simfukwe, John P. Masalu, Najat F. Kahamba, Ismail H. Nambunga, Dickson S. Msaky, Alex J. Limwagu, Abdallah R. Kipekepeke, Carsten Wergin, Rukiyah M. Njalambaha, Elison E. Kemibala, Amour Seleman, Yeromin P. Mlacha, Marceline Finda, Uli Beisel, Esther G. Kimaro, Halfan S. Ngowo, Fredros O. Okumu

**Affiliations:** 1 School of Life Sciences and Bioengineering, The Nelson Mandela African Institution of Science and Technology, P.O.BOX 447, Arusha, Tanzania; 2 Environmental Health and Ecological Sciences Department, Ifakara Health Institute, P.O.BOX 53, Ifakara, Tanzania; 3 Port Health Services Unit, Ministry of Health, Dodoma, United Republic of Tanzania; 4 President’s Office Regional Administration and Local Government, P.O.BOX 528, Mtwara, Tanzania; 5 School of Biodiversity, One Health & Veterinary Medicine, University of Glasgow, Glasgow, G12 8QQ United Kingdom; 6 Heidelberg Centre for Transcultural Studies, Heidelberg University, Voßstraße 2, 69115 Heidelberg, Germany; 7 Muheza College of Health and Allied Sciences, P.O. BOX136, Muheza, Tanzania; 8 Department of Geography, Department of Geography, Free University Berlin, Malteserstr. 74-100, 12249 Berlin, Germany

**Keywords:** *Aedes aegypti*, Mosquito-borne, Habitat characterization, Malaria, Insecticide susceptibility, Tanga port, Tanzania

## Abstract

**Background:**

Increased global trade, while beneficial economically, can also increase the spread of vector-borne diseases, particularly those transmitted by *Aedes* mosquitoes spreading via trade routes. Given the heightened trade-induced activity at ports of entry, it is particularly crucial to assess the risk of mosquito-borne diseases in these settings. This study compared the risks of *Aedes*-borne disease in and around the eastern Tanzanian seaport of Tanga.

**Methods:**

A 200 m × 200 m grid-based system was used to sample mosquitoes within the port area, and in surrounding areas at 2 km, 2.5 km, and 5 km away, between June and December 2023. We characterized mosquito breeding habitats, collected mosquito larvae using standard dippers and tested susceptibility of raised adult *Aedes aegypti* populations to different insecticides. Adult mosquitoes were collected using BG sentinel traps (daytime) and Centers for Disease Control (CDC) light traps (night-time). Additionally, more than 200 port users and neighboring residents were surveyed to assess their experiences with and perceptions of mosquito biting and disease risks.

**Results:**

There were 2931 breeding sites, with (60.8%, *n* = 1782) positive for *Aedes* larvae. The percentage of water-holding containers infested with *Aedes* immatures, i.e., the container index (CI), was highest in the port area (66.2%), and lowest 5 km away (44.6%). The port area also had a greater proportion of temporary breeding sites (64.9%) than did the surrounding areas. The adult mosquito surveys revealed 20,449 mosquito species including: *Culex quinquefasciatus* (56.2%), *Mansonia uniformis* (38.6%), *Ae. aegypti* (5.1%), *Anopheles gambiae* (0.1%), and *Anopheles funestus*. *Ae. aegypti* were more abundant in the port area than in the surrounding areas (*P* < 0.001), whereas *Culex* sp., and *Mansonia* sp., were significantly outside (*P* < 0.001). Adult *Anopheles* sp., were found only in the port area, but *Anopheles* larvae were found both within and outside the port areas. Tests on *Ae. aegypti* sp., revealed susceptibility to bendiocarb and DDT, and resistance to permethrin. Awareness of mosquito-borne diseases among respondents was high for malaria (64.8%), but low for dengue (26.3%) and Chikungunya (1.7%). Most respondents reported being bothered by mosquitoes mostly at night (53.4%) or in the evening (40.7%). In addition to insecticidal bednets, which are used primarily against malaria, preventive measures for *Aedes*-borne diseases are limited.

**Conclusions:**

This study identified significant potential risk of *Aedes species*, specifically *Ae. aegypti* sp., and associated diseases, but low perception of risk and inadequate personal protection measures in the study area. This low perception of risk highlights the need to improve public knowledge of the transmission and control of *Aedes*-borne diseases.

**Graphical Abstract:**

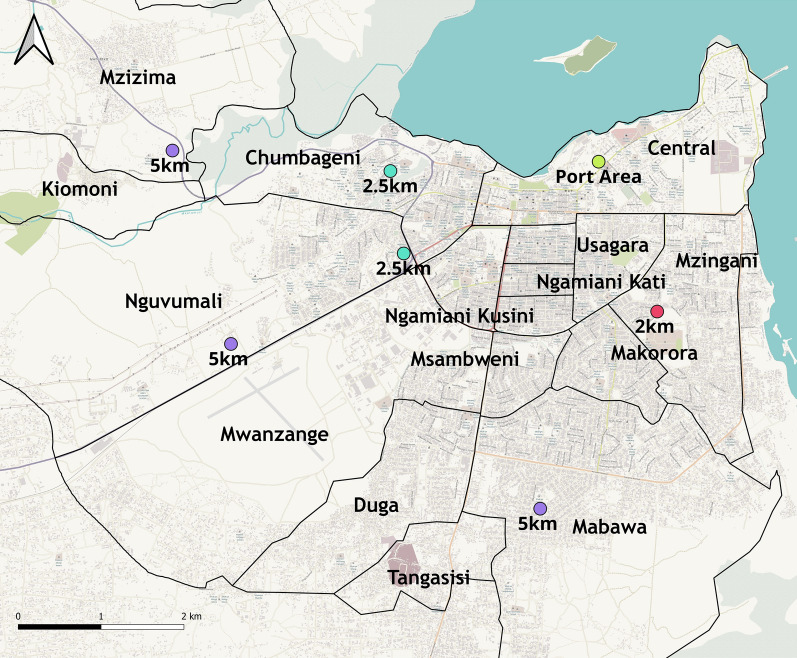

**Supplementary Information:**

The online version contains supplementary material available at 10.1186/s13071-024-06586-x.

## Background

In urban Africa, mosquito-borne diseases including dengue, chikungunya, and yellow fever are becoming a major health issue [1]. In addition to malaria, which causes 250 million cases and more than 600,000 deaths annually [2], the World Health Organization (WHO), has indicated that global dengue incidence rates have also been surged dramatically in recent years [3, 4]. The official WHO estimates suggest that 390 million infections occur annually, of which 96 million manifest clinically, even though only a very small proportion of these cases are officially recorded and reported [5]. It has also been estimated approximately 3.9 billion people across 128 countries are at risk of dengue infection [6]. This extensive burden, the multiplicity of mosquito-borne diseases—with half of the world’s population at risk of two or more of the diseases [7] and the close correlation with human factors such as urbanization and trade [8, 9], all underscore the urgent need for effective surveillance and control measures to mitigate the impact of dengue, especially in vulnerable regions.

In Tanzania, while the burden of malaria is closely tracked by research groups and the Ministry of Health, [10–12] the full burden of other mosquito-borne diseases, notably *Aedes*-borne diseases is largely unknown, except for isolated research activities and reviews. For example, while dengue fever is known to occur in Tanzania in all four serotypes alongside Chikungunya and potentially other *Aedes*-borne diseases [13, 14], the overall burden of dengue in the whole country remains uncertain. One previous review indicated that seroprevalence estimates range from 11% to 50.6% [15], and that rates can be particularly high among patients presenting themselves with fever to health facilities. Despite repeated outbreaks in recent years, comprehensive data on the epidemiology of dengue or the ecology of its vector species are very limited, although there is a growing interest in *Aedes* studies in the country [16–19]. This gap in knowledge is particularly concerning given the potential for dengue outbreaks to exacerbate public health challenges in a country already burdened by other mosquito-borne diseases such as malaria. Moreover, ecological, environmental and climate change concerns such as excessive temperature and frequent flooding [20], as well as poor waste management and uncontrolled water storage practices in urban areas [21, 22], will likely increase the burden and potentially disrupt health services. Indeed, it is now estimated that with warming climate, the dengue vector mosquitoes are expected to survive these warmer temperatures better than malaria vectors are expected to survive [23], meaning that Africa might see significant increases in *Aedes*-borne diseases as a direct result of climate change.

The ecology of *Aedes* mosquitoes is indeed intricately linked to urban environments, where they thrive in a variety of habitats especially human-made containers [24]. *Aedes aegypti*, the primary vector for dengue, chikungunya, and Zika, prefers to breed in artificial containers such as discarded vehicle tires, plastic containers, flowerpots, and water storage tanks [25], commonly abundant in urban settings. Like other container-breeding mosquito species, *Ae. aegypti* can thrive almost independently of rainfall seasons, as they exploit water sources that are continuously available in human communities. This ability allows them to maintain their population densities year-round, and therefore presents a persistent risk of disease transmission. Despite the clear importance of understanding *Aedes* ecology for public health, surveys and studies on these mosquitoes remain limited in Tanzania, even in large cities and small towns.

Global travel, trade, and urbanization are key drivers of the spread of vector-borne diseases [26], with ports playing a critical role in the dissemination of mosquito vectors such as *Ae. aegypti*, *Ae. albopictus* [27–29], and *Culex* species [30]. These vectors often establish themselves in port areas, where invasions tend to be higher due to abundant breeding habitats [31–33]. Ports along the Indian Ocean are particularly vulnerable, with recent invasions of *Anopheles stephensi* [34], *Ae. albopictus*, and re-invasions of *Ae. aegypti* being documented [35–38]. The WHO recommends vector surveillance at ports to keep mosquito densities below national thresholds in line with the International Health Regulations (IHR) [39], but many sub-Saharan African countries, including Tanzania, face challenges such as inadequate surveillance infrastructure [40]. A 2019 dengue outbreak along Kenya’s coast, linked to the DENV-3 serotype from Pakistan, demonstrates the risks posed by global travel [41], while studies in Tanzania indicate a serotype shift from DENV-3 to DENV-1 between 2017 and 2019, increasing the risk of severe symptoms due to antibody-dependent enhancement [42]. Surveys also show widespread transmission of all four dengue serotypes and chikungunya inland [43], highlighting the need for comprehensive vector surveillance in ports.

It is also widely recognized that community awareness and preparedness play a crucial role in the control and prevention of mosquito-borne diseases. In Tanzania, it is often assumed that there is already a high degree of awareness of dengue and other mosquito-borne diseases among communities due to extensive public health campaigns and education efforts [44]. However, knowledge about *Aedes*-borne diseases, such as dengue, chikungunya, and yellow fever, has not been adequately investigated. Yet insufficient knowledge poses a significant challenge for effective disease control, as communities may not take the necessary precautions to protect themselves from *Aedes* mosquitoes. Enhancing public awareness about *Aedes*-borne diseases and improving community preparedness through targeted education and outreach programs are essential steps in mitigating the risks posed by these diseases. This is particularly important in port areas, where the influx of travelers and goods increases the potential for disease spread.

The objective of this current study was to assess and compare the risks of disease-transmitting mosquitoes within the eastern Tanzanian seaport of Tanga and its surrounding urban areas along the Indian Ocean focusing especially on *Ae. aegypti* mosquitoes. We sought to establish the mosquito species composition, abundance, insecticide resistance status and their aquatic breeding preferences; and to use this information to evaluate and address the risks of mosquito-borne infections at this critical point of entry. Additionally, we assessed the perceptions of port users and residents of the surrounding urban areas to understand how such perceptions might inform effective control strategies including social and behaviour change communication strategies.

## Methods

### Study area

**Fig. 1:**
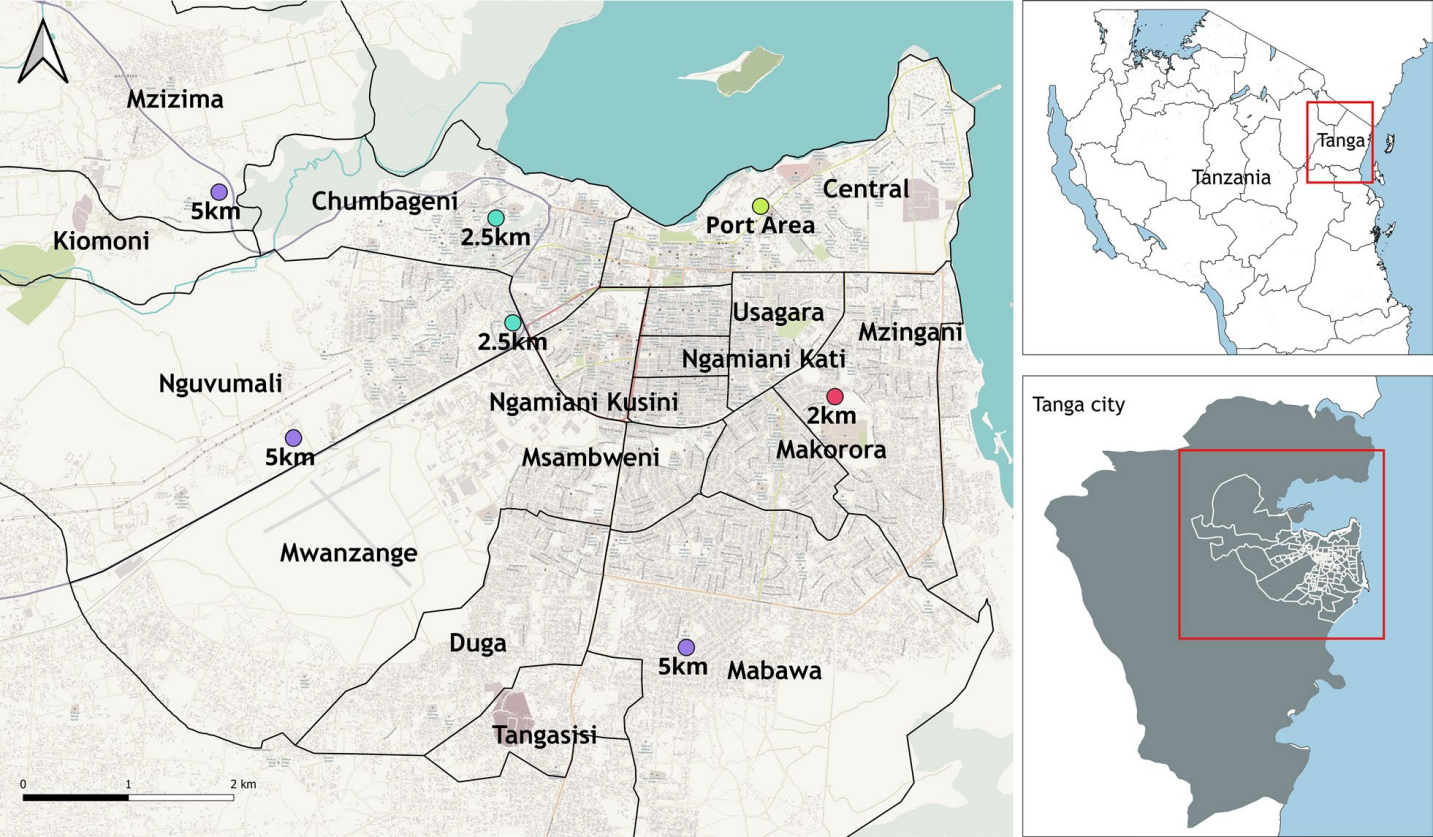
Map of study areas, showing the port area and surrounding wards. Map of the study area contains information from OpenStreetMap contributors, available under the Open Database License. https://help.openstreetmap.org/questions/83255/how-do-i-cite-osm-in-an-academic-paper

The study was conducted in the northeastern Tanzanian port city of Tanga (Fig. 1). The central location was the Tanga seaport, with additional sampling in eight surrounding wards within the Tanga City Council: Central (5° 04′ 19.26'' S, 39° 06′ 06.59'' E), Chumbageni (5° 04′ 14.66'' S, 39° 05′ 21.30'' E), Kiomoni (5° 03′ 13.24'' S, 38° 56′ 57.24'' E), Mzizima (5° 02′ 51.26'' S, 39° 02′ 37.72'' E), Nguvumali (5° 04′ 40.80'' S, 39° 04′ 39.87'' E), Mnyanjani (5° 05′ 56.22'' S, 39° 07′ 13.79'' E), Ngamiani Kusini (5° 05′ 00.42'' S, 39° 06′ 06.14'' E), and Magaoni (5° 06′ 12.49'' S, 39° 06′ 07.06'' E). Tanga District is a border district in the north-eastern part of Tanzania. It is bordered by the Mkinga District Council to the north, the Indian Ocean to the east, Muheza District to the south and west, and the country of Kenya to the north. The district seat is the city of Tanga, which also serves as the administrative and economic center of the region, major economic activities in this area including business, industrial activities, tourism, fishing and agriculture. According to the 2022 census, Tanga District had a total population of just fewer than 400,000 people and some ~100,000 households.

The Tanga seaport is the second largest international point of entry in Tanzania. It was the focal point of the study, and is located in the Central ward, which has an area of ~3.8 km^2^ and an altitude of 10 m above sea level. All sampling sites across all the 8 wards located within 5 km of the port area (Fig. 1).

### Sampling design and site selection

A cross-sectional entomological survey was conducted between June and December 2023 (the dry season). The reference sampling was within the Tanga seaport in the Central ward (within 500 m of the seaport) but additional surveys were conducted at purposively selected sites 2.5 km and 5 km away from the port along three main roads: Pangani, Segera, and Mombasa Road, to understand mosquito distribution across the study area and expansion away from the port. An extra comparison site was selected 2 km south of the port area in the Mnyanjani ward. All the sites were located in the Tanga City Council (Fig. 1).

Sampling was conducted using a 200 m × 200 m grid-based system created in ArcGIS 11.1 environment (ESRI, USA), following the methodology used in a previous *Aedes* survey by Kahamba et al. [16]. Preference was given to grids with human habitation and a total of 23 grids used for the sampling. Each grid was assigned a unique identifier (grid ID) consisting of letters and numbers, with letters representing longitudes and numbers representing latitudes (e.g., A1, A2). The randomization and grid selection procedures were consistent across all locations. An initial survey of the study area was conducted with the assistance of volunteer community resource persons. During the survey, features conducive to mosquito breeding, such as water wells, drains, containers, pots, flowerpots, tins, waste collection points, discarded tires, and drums, were identified. The coordinates of these features were recorded using a handheld GPS (Magellan eXplorist GC, USA). In the central reference area around the Tanga seaport, all the grids within a 500 m buffer zone were selected but no data were collected inside the shipping vessels.

### Sampling of adult mosquitoes

Adult mosquitoes were collected using two types of traps: BG-Sentinel traps or *Aedes* species, and Centers for Disease Control (CDC) light traps for *Anopheles* mosquitoes and culicines.

Five CDC light traps were deployed indoors in selected bedrooms within each grid at night. The traps were set from 18:00 PM to 6:00 AM for six consecutive days before being moved to the next grid in a random fashion. Mosquitoes were retrieved daily from 7:00 AM to 10:00 AM. The retrieved mosquitoes were sorted and identified using taxonomic keys, then preserved in Eppendorf^®^ tubes with silica gel. Concurrently, the BG-Sentinel traps, which were used for outdoor and day-time collections, were positioned in shaded areas in strategic locations near waste collection points, houses, garages, night clubs, and other areas considered to have high mosquito activity zones, and the traps were supplemented with BG lure and molasses mixed with yeast to increase mosquito attraction. Each grid had four traps operating from 6:00 AM to 18:00 PM the following morning for six consecutive days. Mosquitoes were retrieved from the traps daily from 18:00 PM to 19:00 PM, sorted, and identified using a dissecting microscope and taxonomic keys as described above.

For each collection, either by CDC light traps or BG Sentinel Traps, the additional metadata recorded included the grid ID, date, distance from port area, GPS location, and house number. After the samples were sorted, all *Anopheles* and *Aedes* species mosquitoes were sent to Ifakara Health Institute for further analysis.

### Sampling of immature mosquitoes and characterization of their aquatic habitats

Surveys for immature mosquitoes and characterization of breeding habitats were conducted in all selected grids where adult mosquito trapping was being conducted. The search for mosquito larvae focused on various natural and human-made water-holding objects, including used tires, surface drains, wells, discarded containers, animal feeding containers, flowerpots, and buckets. Additional habitats such as tree holes (holes on tree stems), discarded boots, plant leaves, and other small objects that could hold water for more than three days were also examined. Breeding sites with all mosquito larvae or pupae were geo-referenced using handheld GPS devices. The breeding sites were further characterized by assessing the habitat type, location (i.e., present inside the houses or building or outdoors), algae quantity, water color, presence of vegetation, water movement, water source, habitat mobility, and the surrounding environmental and social activities. All information was recorded on breeding site characterization forms.

To collect the mosquito (larvae and pupae) we used a 350 ml dipper or a smaller 70 ml dipper for smaller habitats as adopted from Claudia [45]. The collected larvae and pupae were placed in white trays for morphological identification using pictorial keys [46–48]. The immature mosquitoes were sorted, counted, and data recorded by habitat type, location, and date.

### Rearing of *Aedes* Aegypti larvae for susceptibility testing

After the mosquito larvae were collected in the field, the mosquito species were separated such that only mosquitoes belonging to *Aedes species* were only selected*Aedes* larvae were reared to adulthood for susceptibility testing to establish resistance profiles. Collected immatures were maintained to the adult stage using water from their known breeding habitats mixed with tap water in plastic containers. For rearing, the immatures were placed in labeled basins and were fed with Tetramine® baby fish food. The water was changed every three days to facilitate growth. The rearing was conducted at 26 °C ± 2 °C and 82% ± 10% relative humidity. Pupae were collected daily, counted, and transferred to 30 cm × 30 cm × 30 cm net cages. Emerged adults were fed with 10% glucose and identified morphologically using identification keys [49].

### Testing for susceptibility status of *Aedes aegypti* to insecticides

The susceptibility tests were conducted according to the WHO guidelines for *Anopheles* mosquitoes [50] since we did not have access to appropriate insecticide-impregnated papers for use on *Ae. aegypti*. Adult female *Ae. aegypti* were obtained from the rearing cages to be used for these tests after 3–5 days. The mosquitoes were tested against commonly used insecticides, including pyrethroids (deltamethrin at 0.05% and 0.5% doses and permethrin at 0.75% and 3.75%), an organophosphate (pirimiphos-methyl at 0.25% and 1.25% doses), an organochlorine (DDT 4%), and carbamates (bendiocarb 0.5% and 1%). The higher doses for pyrethroid insecticides were included so as to test the intensity of resistance.

In each bioassay experiment, 120 mosquitoes were distributed into six holding tubes, with 20 mosquitoes per tube. Four tubes were exposed to the test insecticide, and two tubes were served as controls. Mosquitoes in the control tubes were exposed to untreated filter papers, whereas those in the treatment tubes were exposed to filter papers impregnated with the respective doses of the candidate insecticides. The test and control mosquitoes were observed for 60 min. After this, the mosquitoes were transferred back to holding tubes, provided with 10% glucose solution, and then maintained for a day at 28 °C ± 1 °C and 80% ± 10% relative humidity. Mortality was assessed 24-h post-exposure in both the control and treatment arms. A mosquito was considered alive if it could fly, regardless of the number of legs remaining; or dead if they were immobile, unable to stand or take off or obviously moribund.

### Survey of the opinions and perceptions of port users and residents of neighboring wards

To complete the entomological surveys, a comprehensive quantitative survey was conducted to gauge the opinions and perceptions of port users and residents of the neighboring wards regarding *Aedes* mosquito-related risks and disease transmission at entry point and in their areas of residence. We deployed the survey via the *Kobo Collect* toolkit programmed in PC tablets and used a structured questionnaire for the interviews. All participants were recruited voluntarily and were all aged 18 years and above. They included locals, port workers, office staff, entrepreneurs, passengers, drivers, security guards, market vendors, and fishermen, representing diverse groups across the study areas.

Recruitment was performed by visiting workplaces and homes and participants were recruited and surveyed to understand their views on mosquito-related risks at the points of entry. A total of 236 participants voluntarily consented and were recruited. The structured questionnaire assessed key issues such as (i) knowledge of diseases transmitted by mosquitoes in the Tanga port area and surrounding wards, (ii) opinions on groups at greater risk of infection, (iii) mosquito biting experiences, and (iv) personal protection methods used against *Aedes* mosquito bites. Responses were analyzed to identify trends on the basis of occupational status and education levels, providing valuable insights into community awareness and preparedness regarding mosquito-borne diseases.

### Statistical analysis and presentation

Analysis of the quantitative data was conducted using open-source software, R version 3.6.2 [51], and ArcGIS [52]. The data were initially entered into MS Excel and then exported to R in a *csv* format for cleaning and coding. Initially, a descriptive analysis was performed, where we calculated the abundance and distribution of mosquitoes (both adult and immature stages) across sampling locations. A generalized linear model (GLM) with Poisson variate was then employed to determine the associations of mosquito larvae in breeding habitats with habitat characteristics such as water color and movement, the presence of vegetation, and whether the habitat was natural or artificial included as the main predictors. Another GLM with a negative binomial distribution was used to assess the relative comparison of mosquito abundance between sampling areas, trap types, and indoor-outdoor settings at the point of entry, with the grid ID as a random variable to identify significant factors.

To assess the *Aedes* insecticide resistance profile, mosquito populations were classified as susceptible if the mortality rate was greater than 98%, suggestive or unconfirmed resistance that need further investigation if between 90 and 98%, and resistant if less than 90% [50]. All the visualization and spatial analyses was performed in the GIS Environment using QGIS software to analyze mosquito species diversity, abundance, and distribution by interpolation techniques. The environmental data, such as the number of mosquitoes per species, location of collection, and grid ID, trap type, were extracted and compared with *Cx. quinquefasciatus*, *Ma. uniformis* and *Ae. aegypti* abundance. Hotspot analysis using inverse distance weighted (IDW) interpolation was used to identify grids with high mosquito abundance, which were statistically significant and crucial for targeting control measures. IDW was chosen as interpolation method since it estimates cell values by averaging the values of sample data points in the neighborhood of each processing cell. The data were interpolated by means of IDW and transformed from vector into raster.

Lastly, the questionnaire data from the survey of port users and residents were analyzed using descriptive statistics to summarize the participants' knowledge, opinions, and practices regarding mosquito-related risks and disease transmission.

## Results

### Diversity and abundance of adult mosquitoes in the port area and surrounding wards

A total of 20,449 mosquitoes were collected between June and December 2023 (Table 1). Among these: 56.2%, *n* = 11,488 were identified as *Culex quinquefasciatus*, 38.7%, *n* = 7919 as *Mansonia uniformis*, 5.1%, *n* = 1042 as *Aedes Aegypti*, 0.1%, *n* = 16 as *An. gambiae* s.l., and 0.0%, *n* = 3 as *An. funestus* s.l. Outdoor collections using BG sentinel traps accounted for 53.8%, *n* = 11,028, of the total, while indoor collections using CDC light traps comprised 46.1%, *n* = 9421.

**Table 1:** Mean number of mosquitoes caught at different distances from the port area using the CDC light traps (placed indoors for nighttime collections) and the BG sentinel taps (placed outdoors for daytime collections)

Species	Distance (km) from port	CDC light traps (indoors)				BG sentinel traps (outdoors)			
		Total	Mean [95% CI]*	RR [95% CI]	*P* values	Total	Mean [95% CI]*	RR [95% CI]	*p* values
*Culex quinquefasciatus*	0 (port area)	1932	8.99 [7.10, 11.37]	1		1938	5.63 [4.72, 6.73]	1	
	2	856	14.27 [9.17, 22.20]	1.59 [0.96, 2.62]	0.070	1035	10.78 [7.75, 15.00]	1.91 [1.32, 2.79]	< 0.0001
	2.5	1,279	9.84 [7.27, 13.31]	1.10 [0.75, 1.61]	0.643	2,234	10.74 [8.58, 13.44]	1.91 [1.43, 2.54]	< 0.0001
	5	652	5.02 [3.69, 6.82]	0.56 [0.38, 0.82]	< 0.01	1,562	7.51 [5.99, 9.42]	1.33 [1.00, 1.78]	< 0.05
*Aedes aegypti*	0 (port area)	14	0.065 [0.04, 0.12]	1		682	1.98 [1.49, 2.64]	1	
	2	0	0	NA		41	0.43 [0.23, 0.78]	0.22 [0.11, 0.42]	< 0.0001
	2.5	6	0.046 [0.02, 0.11]	0.71 [0.24, 2.06]	0.526	160	0.77 [0.52, 1.14]	0.39 [0.24, 0.63]	< 0.0001
	5	3	0.023 [0.01, 0.08]	0.35 [0.09, 1.34]	0.127	136	0.65 [0.44, 0.97]	0.33 [0.20, 0.54]	< 0.0001
*Mansonia uniformis*	0 (port area)	1129	5.25 [3.45, 7.99]	1		1411	4.10 [2.98, 5.65]	1	
	2	1	0.02 [0.002, 0.14]	0.01 [0.00, 0.03]	< 0.0001	14	0.45 [0.07, 0.32]	0.04 [0.02, 0.08]	< 0.0001
	2.5	2,179	16.76 [9.81, 28.64]	3.19 [1.62, 6.30]	< 0.0001	1231	5.92 [3.93, 8.91]	1.44 [0.86, 2.43]	0.167
	5	1,370	10.54 [6.16, 18.02]	2.00 [1.02, 3.97]	< 0.05	584	2.81 [1.87, 4.25]	0.66 [0.41, 1.16]	0.155

RR, relative risk; CI, confidence interval

* Predicted means from Generalized Linear Model

Most of *Ae. aegypti* mosquitoes were collected outdoors (97.8%, *n* = 1019) on the other hand, half of the *Cx. quinquefasciatus* (58.9%, *n* = 6769) were outdoors. Full summaries are found in Table 1. The highest abundances of *Aedes* mosquitoes collected outdoor were observed in the port area, with populations significantly declining in surrounding areas. Outdoor bite of *Aedes Aegypti* was much lower at 2 km (*z* = −4.47, *p* < 0.001), 2.5 km (*z* = − 3.84, *p* < 0.001) and 5 km (*z* = − 4.45, *p* < 0.001) distance compared with the port area (0 km), as seen in Table 1, with non-linear trends relative to port area. A total of 37 blood-fed *Ae. aegypti* were obtained from port area and 2 at 5 km while none were captured at 2 and 2.5 km distance from the port.

For *Cx. quinquefasciatus*, the indoor bite was lower at 2 km (*z* = 1.81, *p* = 0.07), 5 km (*z* = −2.96, *p* < 0.01) distance compared to the port area (0 km), though bites were the same at 2.5 km (*z* = 1.81, *p* = 0.643), as seen in Table 1. Outdoor bite of *Cx. quinquefasciatus* was found to be lower at 2 km (*z* = 3.39, *p* < 0.001) and 5 km (*z* = 1.96, *p* < 0.05), but higher at 2.5 km (*z* = 4.42, *p* < 0.001) compared with the port area (Table 1).

For *Ma. uniformis* very low indoor collection was found at 2 km (*z* = −5.24, *p* < 0.01) and higher collections at 2.5 km (*z* = 3.34, *p* < 0.001) and 5 km (*z* = 2.00, *p* < 0.05) compared with the port area (Table 1). On the other hand, lower catches were observed at 2 km (*z* = −7.64, *p* < 0.001) while similar catches were observed at 2.5 km (*z* = 1.38, *p* = 0.167) and 5 km (*z* = −1.42, *p* = 0.155) compared with port area for outdoor.

### Spatial distribution of adult *Aedes* mosquitoes

**Fig. 2:**
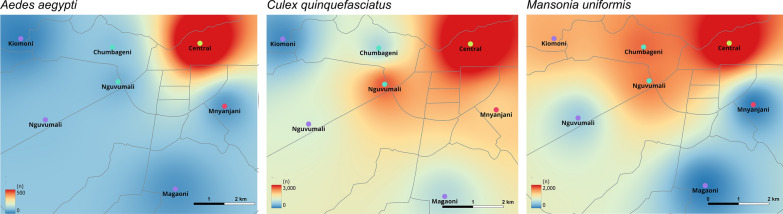
Comparison of mosquito catches in the port area and the surrounding area

The distribution of adult *Aedes* mosquitoes varied across the surveyed administrative wards. The results from interpolation techniques by inverse distance weighted (IDW) interpolation of a point vector layer revealed a higher density of *Aedes* mosquitoes in the port area located in the Central ward, indicating these as hotspots. Conversely, areas such as Mnyanjani (2 km from the port), and Magaoni and Kiomoni (5 km from the port) were identified as having lower densities of *Aedes* mosquitoes as shown in Fig. 2.

### Diversity and abundance of immature stages and their aquatic habitats in the port area and surrounding wards

The larval searches and habitat characterization activities identified immature stages of *Anopheles*, *Culex*, and *Aedes* mosquitoes in several habitats across the study area. Out of 2934 sampled mosquitoes, 60.7% were *Aedes*, 39.1% were *Culex*, and 0.1% were *Anopheles* (Table 2). High densities of *Aedes* immatures were found in Central ward within the port area (68.3%), followed by Nguvumali ward (11.9%), with the lowest density in Kiomoni ward (1.1%). Notably, *Anopheles* larvae were only detected in Magaoni ward. *Culex* larvae were most prevalent in Central ward (60.4%), followed by Nguvumali (12.9%).

**Table 2:** Sampled populations of *Aedes, Anopheles* and *Culex* larvae

Wards	*Aedes*	*Culex*	*Anopheles*	Total
	*N* (%)	*N* (%)	*N* (%)	*N* (%)
Central (location of port)	1,21,768.3	69,460.4	00	1911
Chumbageni	1327.4	776.7	00	209
Kiomoni	201.1	847.3	00	104
Magaoni	522.9	282.4	4100	84
Mnyanjani	693.9	363.1	00	105
Mzizima	321.8	564.9	00	88
Ng/Kusini	472.6	262.3	00	73
Nguvumali	21,211.9	14,812.9	00	360
Total	1781 (60.7%)	1149 (39.1%)	4 (0.1%)	2934 (100%)

N, number of larvae collected, and %, percentage of larvae by ward surveyed

### Larval indices—for *Aedes* mosquitoes

A total of 2931 breeding sites were identified in 416 households. Of these, 60.8% (1,781/2,931) were positive for *Aedes* immatures stages. Various indices were calculated, including the container index (CI), house index (HI), and Breteaux index (BI). The highest CI values were observed in the port area (66.2%), 2.5 km away (51.5%), and 5 km away (44.6%). For the HI, the highest values were at 5 km (61.5%) and 2 km (60%) from the port, while the port area had the lowest HI (44%). Lastly, the highest BI values were found at 2 km (45.7%) and 5 km (30.3%) from the port, with the port area having the lowest BI (7.7%), as shown in Table 3.

**Table 3:** Summary of *Ae. aegypti* larval survey indices

Name of area	No. of premises	No. of positive premises	House index (HI)	No. of containers surveyed	No. of positive containers	Container index (CI)	Breteaux index (BI)
Port area	150	66	44	1748	1158	66.2	7.7
2 km away	35	21	60	195	16	8.2	45.7
2.5 km away	122	66	54.1	68	35	51.5	28.9
5 km away	109	67	61.5	74	33	44.6	30.3

Container Index (CI), ratio of larval infested containers to total inspected containers; house index (HI), ratio of larval infested houses to all inspected houses; Breteaux index (BI), ratio of positive containers per 100 houses inspected

### Habitat characterization

During the habitat search for *Aedes* mosquitoes, the breeding sites were characterized on the basis of physical parameters such as algal quantity, water source, watercolor, water movement, water type, and vegetation quantity (Table 4). A total of 2931 breeding sites were identified, of which 22.7%, 664/2931, were used tires, 71.1%, 2085/2931, were containers (both plastic and metal), 1.3%, 38/2931, were surface drains, 4.5%, 132/2931, were flowerpots, and 0.4%, 12/2931, was coconut shells. The majority of positive breeding habitats for *Aedes* mosquitoes had clear water (62.9%, 1,242/1973), moderate vegetation (66.9%, 696/1041), water resulting from rainfall (60.9%, 1529/2525), scarce algal quantity (60.8%, 1033/1698), and stagnant water (61%, 1777/2911). The different habitat types of *Aedes* mosquitoes observed in the study area are shown in Fig. S1 and Fig. S2.

**Table 4:** Physical characteristics of the *Aedes* mosquitoes’ breeding habitats identified in the study area

Parameter	Category	Positive, *n* (%)	Negative, *n* (%)	Total
Habitat type	Disposed containers*	1158 (55.5)	927 (44.5)	2085
	Coconut shell	12 (100)	0	12
	Surface drains	15 (39.5)	23 (60.5)	38
	Flowerpots	100 (75.8)	32 (24.2)	132
	Tires	496 (74.8)	168 (25.2)	664
Algae quantity	None	360 (57.4)	267 (42.6)	627
	Moderate	372 (65.1)	199 (34.9)	571
	Scarce	1033 (60.8)	665 (39.2)	1698
	Abundant	16 (47.1)	18 (52.9)	34
Water source	Domestic	252 (62.2)	153 (37.8)	405
	Rainwater	1529 (60.6)	996 (39.4)	2525
Water color	Clear	1242 (62.9)	731 (37.1)	1973
	Polluted	539 (56.3)	418 (43.7)	957
Water movement	Stagnant	1777 (61.1)	1134 (38.9)	2911
	Slow	4 (21.1)	15 (78.9)	19
Water type	Permanent	530 (52.8)	473 (47.2)	1003
	Temporary	1251 (64.9)	676 (35.1)	1927
Vegetation quantity	None	452 (51.9)	419 (48.1)	871
	Moderate	696 (66.9)	345 (33.1)	1041
	Scarce	454 (61.1)	289 (38.9)	743
	Abundant	179 (65.1)	96 (34.9)	275
Total		1781	1150	2931

(*): The disposed containers included plastic drums, buckets, tins, plastic barrels, basins, metal drums, jerrycans, and tanks

### Association between habitat characteristics and mosquito densities

Disposed containers were the primary habitat for both *Aedes* and *Culex* mosquitoes, with a high number of mosquitoes collected (65.6%; *n* = 1158). Overall, the likelihood of capturing *Aedes* mosquitoes in disposed tires was about three times higher compared to disposed containers (*z* = 7.401, *p* < 0.001, Table 5). *Aedes* species also preferred habitats with scarce algae level (*z* = 1.77, *p* < 0.05), turbid/polluted water (*z* = −2.66, *p* < 0.01), and temporary aquatic habitats (*z* = −9.120, *p* < 0.001, Table 5) with slow moving water (*z* = − 3.35, *p* < 0.01).

**Table 5:** Mean number of mosquitoes collected in different habitat types (and 95% CI)

	Habitat characteristics		Total	Mean ± 2SE*	RR [95% CI]	*p* values
*Aedes* spp.	Habitat type	Disposed containers	1158	4.3 ± 1.0	1	
		Coconut shell	12	2.4 ± 1.2	0.9 [0.6, 2.4]	= 0.846
		Surface drains	15	1.2 ± 0.8	0.8 [0.4, 1.6]	= 0.528
		Flowerpots	100	3.0 ± 1.2	1.3 [0.9, 2.0]	= 0.187
		Tires	496	7.1 ± 2.1	2.8 [2.1, 3.6]	< 0.001
	Algae quantity	None	360	13.0 ± 5.9	1	
		Moderate	372	3.0 ± 0.9	0.6 [0.4, 0.9]	< 0.05
		Scarce	1033	4.5 ± 1.0	0.7 [0.4, 1.0]	< 0.05
		Abundant	16	3.2 ± 2.6	0.6 [0.2, 1.7]	= 0.348
	Water source	Domestic	252	3.1 ± 0.8	1	
		Rainwater	1529	5.0 ± 1.0	0.8 [0.6, 1.1]	= 0.156
	Water color	Clear	1242	5.1 ± 1.0	1	
		Turbid	539	3.7 ± 1.3	0.7 [0.6, 0.9]	< 0.01
	Water type	Permanent or semi-permanent	530	23.0 ± 8.0	1	
		Temporary	1251	3.4 ± 0.5	0.1 [0.1, 0.2]	< 0.001
	Vegetation quantity	None	452	14.6 ± 5.8	1	
		Moderate	696	3.6 ± 0.6	1.1 [0.8, 1.6]	= 0.638
		Scarce	454	3.7 ± 1.5	1.1 [0.6, 2.0]	= 0.746
		Abundant	179	4.2 ± 2.2	0.9 [0.6, 1.3]	= 0.414
*Culex* spp.	Habitat type	Disposed containers	927	3.5 ± 0.8	1	
		Coconut shell	0	0	0.0 [0.0, 0.0]	0.999
		Surface drains	23	1.8 ± 0.6	0.8 [0.4, 1.7]	0.591
		Flowerpots	32	1.0 ± 0.6	0.5 [0.3, 0.8]	< 0.01
		Tires	167	2.4 ± 1.0	1.1 [0.8, 1.4]	0.629
	Algae quantity	None	267	9.5 ± 5.0	1	
		Moderate	199	1.6 ± 0.3	0.5 [0.3, 0.9]	< 0.05
		Scarce	665	2.9 ± 0.7	0.7 [0.4, 1.0]	= 0.067
		Abundant	18	3.6 ± 2.0	1.6 [0.6, 4.5]	= 0.401
	Water source	Domestic	153	1.9 ± 0.7	1	
		Rainwater	996	3.2 ± 0.7	1.3 [1.0, 1.8]	= 0.064
	Water color	Clear	731	3.0 ± 0.8	1	
		Turbid	418	2.9 ± 1.0	1.3 [1.1, 1.7]	< 0.05
	Water type	Permanent or semi-permanent	473	20.6 ± 5.5	1	
		Temporary	676	1.9 ± 0.3	0.2 [0.1, 0.2]	< 0.001
	Vegetation quantity	None	419	13.5 ± 5.0	1	
		Moderate	345	1.8 ± 0.3	0.6 [0.4, 1.1]	= 0.057
		Scarce	289	2.4 ± 0.9	0.6 [0.4, 1.0]	< 0.05
		Abundant	96	2.2 ± 0.5	0.6 [0.4, 1.1]	= 0.095

*2SE; Refers to two standard errors

For the *Culex* mosquitoes, the likelihood of capturing *Culex species* in flower pots was 0.5 times lower compared with disposed containers (*z* = −2.96, *p* < 0.01, Table 5). Similarly, *Culex species* prefer moderate amount of algae (*z* = −2.55, *p* < 0.05, Table 5). Similar to *Aedes*, the *Culex* species also prefer temporary aquatic habitats (*z* = −8.50, *p* < 0.001, Table 5) with polluted water (*z* = 2.05, *p* < 0.05, Table 5). A full detailed summary is found in Table 5.

### Susceptibility of *Aedes aegypti* mosquitoes to public health insecticides

**Fig. 3:**
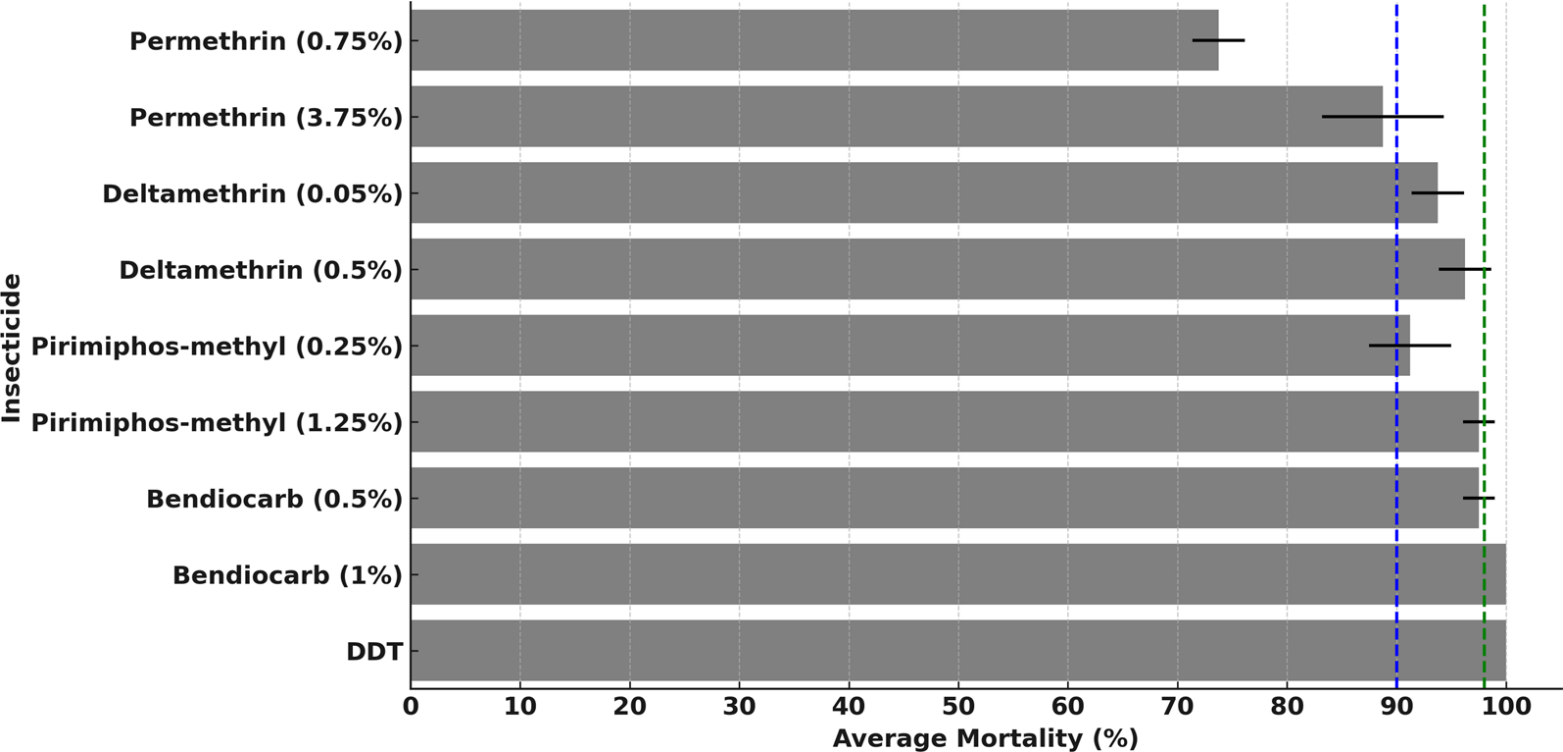
Results of the susceptibility tests for female *Ae. aegypti* mosquitoes showing mean mortality after 24 h of monitoring post-exposure to the candidate insecticides. The dotted green lines (≥ 98% mortality) indicate full susceptibility, while the dotted blue lines (90–98% mortality) indicate possible resistance or unconfirmed resistance requiring confirmation

Susceptibility tests for female *Ae. aegypti* mosquitoes aged 3–5 days were conducted against five insecticides at various concentrations, including permethrin (0.75%, 3.75%), deltamethrin (0.05%, 0.5%), pirimiphos-methyl (0.25%, 1.25%), DDT (4%), and bendiocarb (0.5%, 1%). At 24 h post-exposure, the average mortality rates for *Ae. aegypti* exposed against bendiocarb (1%) and DDT (4%) were 98.8% and 100%, respectively, indicating full susceptibility. The average mortalities associated with pirimiphos-methyl (0.25%) and deltamethrin (0.05%) were 94.4%, 93.8%, respectively, suggesting possible or unconfirmed resistance. Increasing the dose of deltamethrin to 0.5% increased the 24-h mortality to 96.3%, suggesting that even at this dose the mosquitoes were still slightly resistant. Tests against permethrin showed average mortality rates of 73.8% and 88.8%, indicating clearly confirmed resistance even at the five times higher dose of 3.75%. In contrast, the *Ae. aegypti* mosquitoes were fully susceptible to DDT and bendiocarb (Fig. 3).

### Awareness and perceptions of the risk of *Aedes*-borne diseases

#### Demographic characteristics of survey respondents

A total of 236 respondents participated in the survey within the port area and the nearby community (Table 6). Among the participants, 58.1%, *n* = 137, were male and 41.9%, *n* = 99, were female. The majority had a secondary level of education (47.0%, *n* = 111), with the age interval ranging from 36–45 years (36.4%, *n* = 86). Primary economic activities among respondents were self-employment including entrepreneurship (73.3%, *n* = 173), formal employment including government employee (21.2%, *n* = 50), fishing (3.0%, *n* = 7), unemployed (1.7%, *n* = 4), and other were farmers (0.8%, *n* = 2).

**Table 6:** Characteristics of study respondents at Tanga point of entry (*N* = 236)

Category	Variable	*n* (%)
Sex	Female	99 (41.9)
	Male	137 (58.1)
Occupation	Self-employed	173 (73.3)
	Fisherman	7 (3.0)
	Public services (health and other workers)	50 (21.2)
	Farmer	2 (0.8)
	Unemployed	4 (1.7)
Age	16–25	45 (19.1)
	26–35	84 (35.6)
	36–45	86 (36.4)
	46–55	20 (8.5)
	56 and above	1 (0.4)
Education level	Illiterate	3 (1.3)
	Primary	47 (19.9)
	Secondary	111 (47.0)
	University	75 (31.8)
Marital status	Married	131 (55.5)
	Unmarried	104 (44.1)
	Widow	1 (0.4)

### Awareness, perception, and experience of mosquito-borne diseases among residents of Tanga seaport

The survey results revealed that approximately two thirds of the respondent reported that only female mosquitoes bite humans and transmit diseases (69.8%, *n* = 134) while about one third reported that both sexes of mosquito bite human and transmit diseases (27.6%, *n* = 53). Overall, about two-third (64.8%, *n* = 153) of all respondents were aware of mosquitoes transmitting only malaria, and less than one third (26.3%, *n* = 62) were aware that mosquitoes also transmit dengue fever viruses. Chikungunya is far less recognized and was identified as a mosquito-borne disease by only 1.7%, *n* = 4, of respondents.

Approximately 97.9%, *n* = 231, of the respondents perceived that there is a risk of contracting mosquito-borne diseases or infections at the seaport where only 2.1%, *n* = 5, respondents reported no perceived risk. Additionally, about two thirds of all respondents reported that workers at the port are perceived to be at greatest risk of mosquito-borne infections (61.4%, *n* = 145) followed by community living nearby port area (28%, *n* = 67), indicating that people who reside around the seaport area, have highest perception of risk than those living away from the seaport area. Lastly, the majority of the survey respondents reported being bothered by mosquito bites at night (53.4%, *n* = 126) and in the evening (40.7%, *n* = 96). Other than insecticide treated nets (ITNs), which were widely used for malaria prevention, only a smalls percentage of respondents reported using other interventions against mosquito-borne diseases, these other interventions included the use of long clothing (19.1%, *n* = 45), topical repellents (12.3%, *n* = 29), and swatting with bare hands (11.9%, *n* = 28) as shown in Table 7.

**Table 7:** Awareness, perception, and experience of mosquito-borne diseases among residents of Tanga seaport

Question asked	Variables	*n* (%)
Do you know the sex of mosquito that bite human?	Yes	192 (81.4)
	No	44 (18.6)
Which sex of mosquito biting human?	Female	134 (69.8)
	Male	5 (0.6)
	Both sexes	53 (27.6)
Which diseases you know are transmitted by mosquitoes?	Malaria	153 (64.8)
	Chikungunya	4 (1.7)
	Dengue	62 (26.3)
	Malaria, dengue, chikungunya, and others	17 (7.2)
Is there any risk of contracting mosquito-borne diseases or infections while at seaport?	Yes	231 (97.9)
	No	5 (2.1)
Which group is at greater risk of mosquito-borne infection in seaport?	All people	4 (1.7)
	Nearby community	67 (28.4)
	Fishers	2 (0.8)
	Passengers/travelers	13 (5.5)
	Undecided	5 (2.1)
	Workers at port	145 (61.4)
Time at which mosquito bite more while at seaport	Evening	96 (40.70
	Morning	5 (2.1)
	All the time	7 (3)
	Night	126 (53.4)
	Noon	2 (0.8)
Personal protection methods used against mosquito biting at seaport	Swatting	28 (11.9)
	Nothing	134 (56.8)
	Use of long clothing	45 (19.1)
	Use of repellents	29 (12.3)

## Discussion

Most studies on arbovirus vectors in Africa have been reactive to outbreaks or focused on large urban populations, neglecting high-risk areas such as international points of entry, which can be hotspots for mosquito-borne viruses [53–57]. Despite being of significant economic value, international shipping activities at ports can facilitate the migration of vectors, and can pose significant threats in the absence of effective surveillance systems [26]. For this reason, strengthening the basic entomological surveillance systems at all major international points of entry has been recommended both by the IHR [58] and Tanzania’s National Strategy for Vector Control (2019–2024) [40]. The aim of this study was therefore to compare the risks of disease-transmitting mosquitoes, with a focus on *Aedes* mosquitoes within and in the surrounding urban areas of the eastern Tanzanian seaport of Tanga along the Indian Ocean. To complement these entomological surveys, we also assessed the knowledge and perceptions of port users and residents of surrounding wards regarding the risk and control of mosquito-borne infections in the area.

The entomological study identified five mosquito species: *An. gambiae* s.l., *An. funestus*, *Ae. aegypti*, *Cx. quinquefasciatus*, and *Mansonia uniformis*. The high abundance of *Ae. aegypti* in the port area suggests it is a hotspot for this species, which is consistent with findings from other studies in Mumbai International Seaport (India) and the port of Abidjan [31, 59]. The presence of discarded tires and containers likely contributes to this high density. While most *Aedes* mosquitoes were caught in outdoor BG sentinel traps during the day, indicating significant outdoor biting activity, *Culex* species were commonly found both indoors and outdoors. Notably, the low numbers of *Anopheles* mosquitoes in the port area aligned with their ecological preference for rural areas and limited breeding opportunities in urban settings of Tanga as evidenced in previous studies in which malaria vectors, including *An. funestus*, preferred to breed along rivers with slow-moving clear waters and emergent vegetation by *Nambunga *et al. (2020) [60] and swamps or large drain by *Sattler *et al. (2005) in Dar es salaam [61], resulting in low levels of malaria transmission. Overall, the findings of this study highlight the significant presence and distribution of key disease-transmitting mosquitoes, particularly *Ae. aegypti*, within the Tanga port area and its surrounding wards. The high composition of *Ae. aegypti* in the present study correspond with the findings of survey studies in urban regions of (Dar es Salaam, Coast, Tanga and Arusha) by *Philbert *et al. (2020), where the composition was dominated by *Aedes* genera despite the use of traps different from those used in the present study [62].

The *Aedes* species preferred breeding in discarded tires and containers, such as water buckets and drums, which is consistent with findings from earlier studies of deendayal seaport, kandla, gujarat in india, and the state of Maranhao in Brazil [63, 64]. These elevated densities underscore the importance of ports as critical points for mosquito surveillance and control to address the risk of diseases such as dengue, chikungunya, and Zika. Field data from both the port and its surrounding wards clearly revealed that, indeed, *Ae. aegypti* populations decreased with increasing distance from the port, confirming that the port area is likely a hotspot, potentially because of factors such as stagnant water in containers and increased human activity during the day.

Interestingly, while malaria remains the most recognized mosquito-borne disease among the respondents, awareness of dengue and other *Aedes*-transmitted diseases is considerably lower than in the study conducted in Pwani in 2019, where the majority seemed to be aware of dengue fever virus (DFV) [65], the raised awareness about DFV influenced by the major outbreak of dengue occurred in Dar es salaam in 2019 [66].

Malaria is indeed also a significant public health problem in Tanzania though the risk in these urban settings is lower. The latest survey showed that malaria prevalence in Tanga was 4% [11], and in this survey, we found only a negligible number of *Anopheles* mosquitoes in the survey. According to the latest Demographic Health and Malaria Indicator Survey (TDHS and MIS 2022), household bed net use in Tanga was 84.2%, the percentage of children who had fevers in the preceding 2 weeks was 8.5%, and the overall malaria prevalence in children was 4% [11]. It is understandable why communities continue with an emphasis on malaria but the neglect of *Aedes*-borne diseases is particularly concerning given the rising global incidence of dengue and the presence of *Ae. aegypti* in the region [35, 38, 54, 62, 67]. The limited use of personal protective measures against *Aedes* mosquitoes, as indicated by our survey, further exacerbates the risk of Aedes-borne diseases. Other than bed nets, which protect people mostly at night-time, the majority of respondents did not use any form of protection against day-biting mosquito bites such as *Ae. aegypti*, with only a small fraction using methods such as long clothing or repellents. This highlights the urgent need for public health education campaigns to improve awareness and encourage the adoption of protective measures against *Aedes* mosquitoes (day-biters) among port users and surrounding communities.

Susceptibility tests showed that *Ae. aegypti* were susceptible to Bendiocarb, but full susceptibility to DDT was observed, consistent with previous research in Ifakara, Tanzania [16]. However, reduced susceptibility to deltamethrin was detected consistent, which is consistent with the findings of a previous study in Dar Es salaam [68], indicating possible resistance. The potential resistance to deltamethrin in our study may have resulted from repeated chemical use in the area. Additionally, *Ae. aegypti* showed reduced susceptibility to pirimiphos-methyl, contradicting recent studies in Ifakara [16] and Nigeria [69], which reported full susceptibility. These differences might be due to either ecological differences or seasonal variations. Furthermore, the present study confirmed the resistance of *Ae. aegypti* to permethrin, similar to the findings of a previous study in Dar Es salaam [68], one of the most common pyrethroids used in both agriculture and public health in the area. Overall, these investigations show that the available chemical options are increasingly limited and that greater vigilance is required. These tests should therefore be periodically repeated, preferably using more standardized tests with protocols designed specifically for *Ae. aegypti* mosquitoes as recommended by the WHO [70] to best determine the range of options for control.

This study also revealed significant gaps in knowledge among port users and neighboring residents regarding mosquito-borne diseases. While most participants were aware that malaria is a major public health problem, very small proportions, consisting predominantly of people with tertiary education were aware of other mosquito borne diseases such as dengue and chikungunya viruses. This is a common phenomenon in malaria-endemic areas, where awareness of mosquito bites and their role in malaria transmission is commonly found to be relative high, whereas knowledge about arboviral diseases transmitted by *Aedes* mosquitoes is notably limited [71]. For instance, in one study in the Central African Republic, most respondents identified malaria as the sole mosquito-borne disease despite evidence of dengue and other mosquito-borne diseases in circulation [72]. However, as these arboviruses become more significant public health challenges, greater efforts, including social and behavior change communication strategies should be adopted to improve community knowledge of the disease prevention and their control management.

In the assessment of community perceptions regarding groups at risk, a significant proportion of respondents acknowledged that port users and nearby communities are at risk of mosquito bites and infections, even though they did not distinguish between the different diseases. Moreover, people working at the port were considered to be at greater risk than the rest of the communities. The respondents also reported frequent mosquito bites, especially in the evening and at night, highlighting their exposure. This study aligns with previous research indicating that behavioral responses to infection risk are shaped by perceived exposure and bite frequency [73]. Despite recognizing their vulnerability, respondents showed poor adoption of personal protection measures. This mismatch of risk perception and self-protection was a surprise as previous studies have shown a direct correlation of these variables-people with high awareness being correlated with higher engagement in protective behaviors [74].

Although the main objectives were successfully achieved, this study had some limitations. First, the limited number of *Aedes* traps used for collecting adult mosquitoes may not have captured the full distribution effectively. Second, larvae and pupae collections were restricted to selected grids with human occupations or buildings, potentially leading to an underestimation of overall densities and distribution. Additionally, in the tests for insecticide susceptibility, WHO standard doses for *Anopheles* mosquitoes were used due to the lack of appropriate papers for testing *Aedes* mosquitoes. However, some of these insecticides, such as pirimiphos methyl, permethrin, and deltamethrin, already have diagnostic concentrations specific for *Aedes* mosquitoes, whereas other insecticides have different diagnostic concentrations specific for *Aedes* mosquitoes, which if used, might have yielded different results.

## Conclusions

This study highlights the significant risk of *Ae. aegypti* and associated diseases within the Tanga port area, emphasizing the critical role of ports in the surveillance and control of mosquito-borne diseases, particularly those transmitted by *Aedes* mosquitoes. Despite the community being more concerned with malaria, awareness of other mosquito-borne diseases remains limited and should be increased given the observed risk. High abundance of *Ae. aegypti* in the port area, especially in discarded tires and containers, underscores the need for targeted interventions and more broadly, environmental sanitation to reduce the risk. Insecticide susceptibility tests revealed full susceptibility to bendiocarb and DDT but emerging resistance to pirimiphos-methyl and deltamethrin, suggesting that options for effective control may be limited; therefore, there is a need to for diversified control strategies. The study also revealed that the central port area, compared with other sites, is likely a hotspot for mosquito breeding, with *Aedes* populations decreasing with distance from the port. Since this study was the first dedicated mosquito survey to target this international point of entry, the results will form a basis for future research on pathogen transmission and control programs. The identification of key breeding habitats offers potential targets for *Aedes* control, emphasizing the need for integrated vector management involving community engagement, environmental management, and periodic insecticide rotation. The key recommendations are as follows: a) authorities should integrate environmental management, insecticide use, and community engagement to address discarded habitats; b) port health authorities should implement robust *Aedes* mosquito control measures to prevent potential outbreaks; c) future insecticide susceptibility studies should incorporate specific guidelines and appropriate concentrations for *Aedes* mosquitoes; d) port workers and nearby communities should be educated on mosquito control and prevention; and e) year-round studies should be conducted to understand seasonal variations in mosquito densities and resistance profiles. In conclusion, this study identified a relatively high potential risk of *Ae. aegypti* and associated diseases, but a low perception of risk and inadequate personal protection measures in the study area. This low perception of risk highlights the need to improve public knowledge of the transmission and control of *Aedes*-borne diseases.

## Supplementary Information

Supplementary material 1: Fig. S1: Common habitat types observed in the study area: a shallow well, b surface drain c discarded bowl holding water, d root hole of banana tree, e discarded car tires, f fire hydrant

Supplementary material 2: Fig. S2: Common habitat types observed in the study area: a flowerpot, b plastic buckets for animal drinking, c container for feeding hens, d inspection chamber, e plastic container holding water placed under charcoal burner, f stream pool

## Data Availability

No datasets were generated or analysed during the current study.
