# Supplementary figures and images for: Risk of Aedes-borne diseases in and around the Tanzanian seaport of Tanga despite community members being more concerned about malaria
Source: Parasit Vectors. 2024 Dec 18;17:512. doi: 10.1186/s13071-024-06586-x (PMC11657424; doi:10.1186/s13071-024-06586-x)

## Slide 1
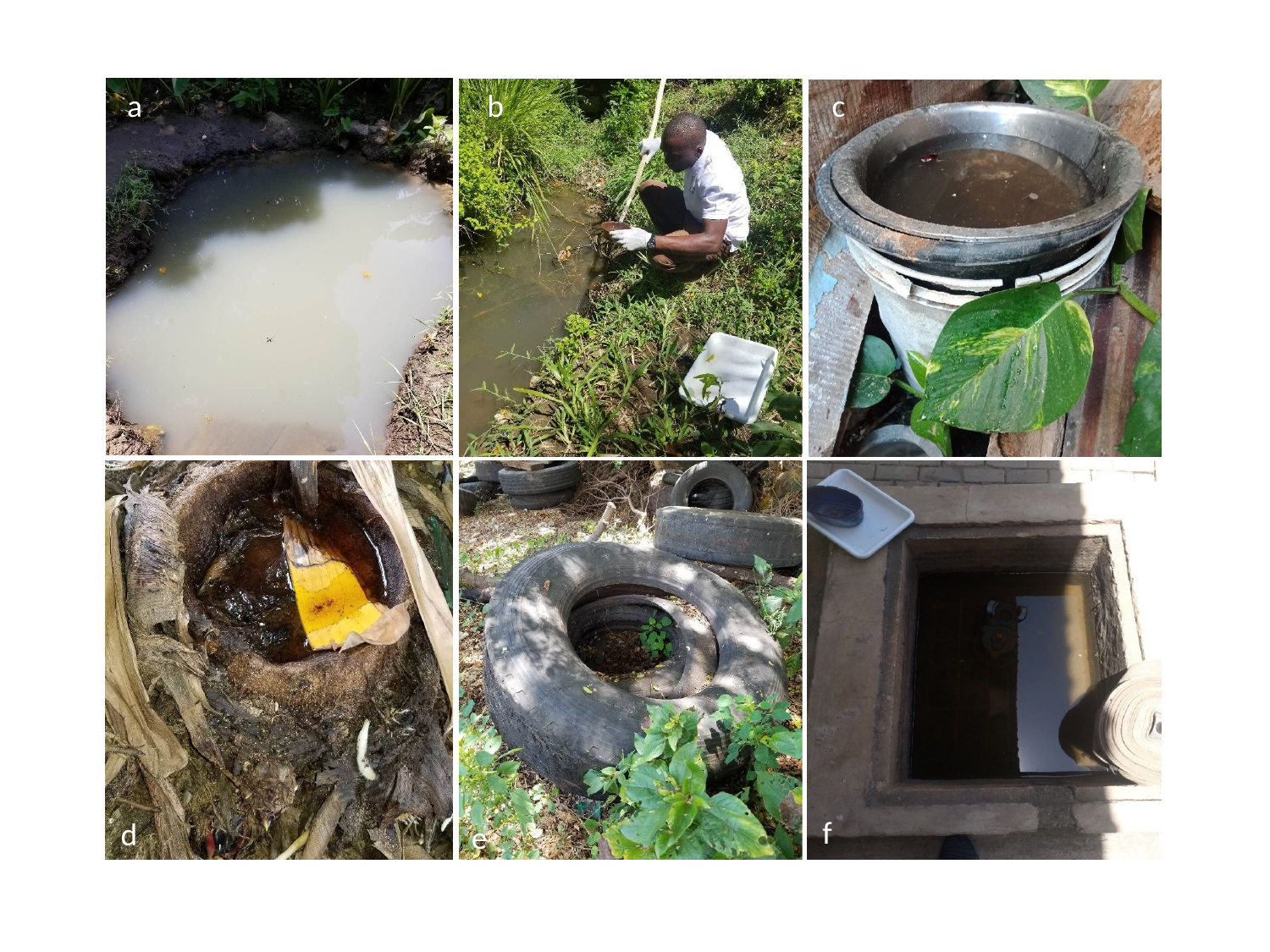

a
b
c
f
d
e

Supplement: Supplementary file 1 — Supplementary material 1: Fig. S1: Common habitat types observed in the study area: a shallow well, b surface drain c discarded bowl holding water, d root hole of banana tree, e discarded car tires, f fire hydrant [file 13071_2024_6586_MOESM1_ESM.pptx]

## Slide 1
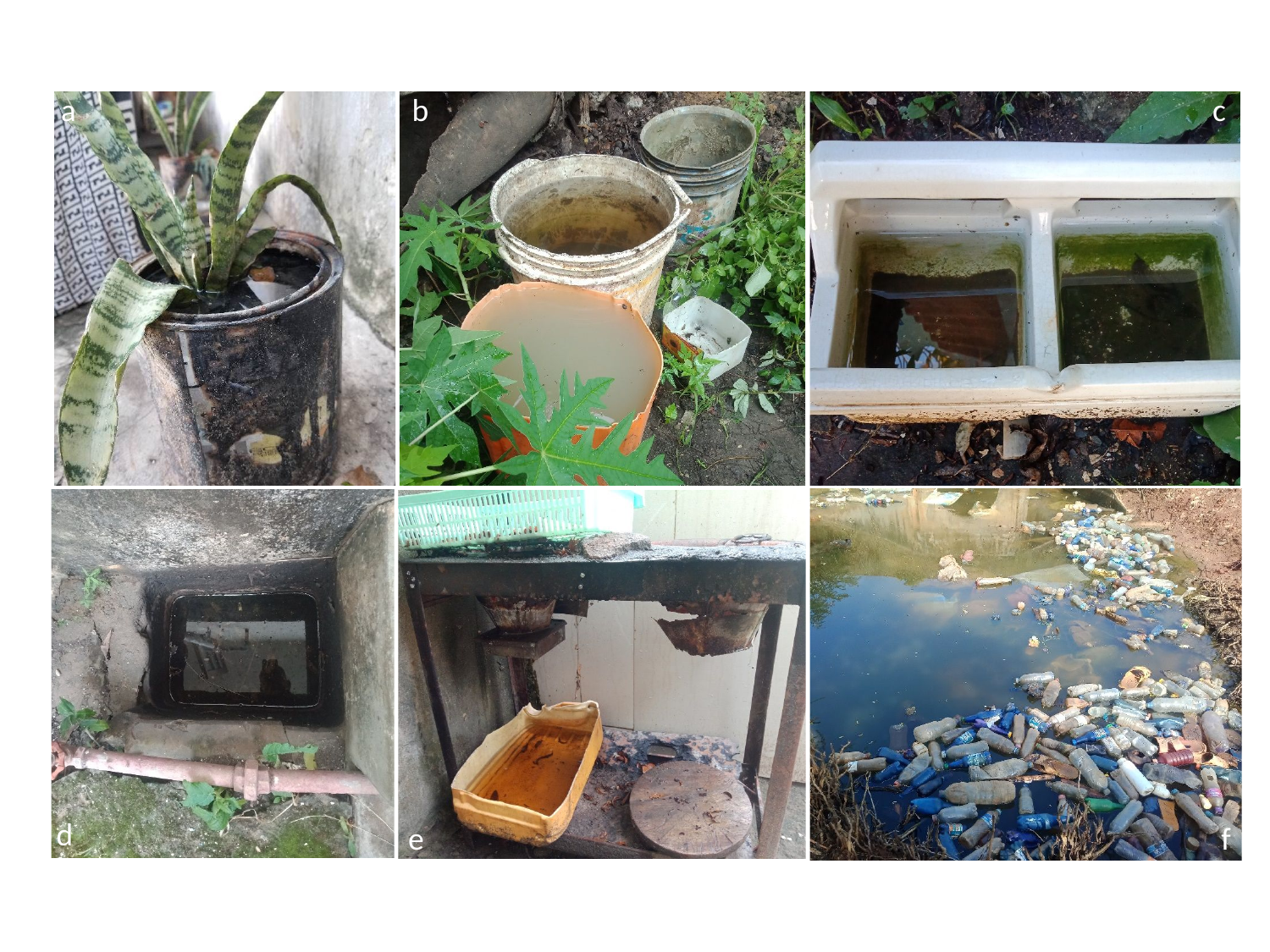

a
b
c
d
e
f

Supplement: Supplementary file 2 — Supplementary material 2: Fig. S2: Common habitat types observed in the study area: a flowerpot, b plastic buckets for animal drinking, c container for feeding hens, d inspection chamber, e plastic container holding water placed under charcoal burner, f stream pool [file 13071_2024_6586_MOESM2_ESM.pptx]
